# Attitudes towards wife-beating justification and its association with female genital mutilation – analysis of ever-married Somali women in the 2020 Somali Health and Demographic Survey

**DOI:** 10.1093/inthealth/ihae047

**Published:** 2024-06-17

**Authors:** Abdirahman Saeed Mohamed, Espen Bjertness, Aung Soe Htet, Win Thuzar Aye, Ahmed Ali Madar

**Affiliations:** School of Graduate Studies University of Hargeisa, Pepsi Road, Ahmed Dhagah District, Hargeisa, Somaliland; Department of Community Medicine and Global Health, Institute of Health and Society, University of Oslo, Post Box 1130 Blindern, 0318 Oslo, Norway; Department of Community Medicine, Faculty of Health Sciences, UiT The Arctic University of Norway, 9037, Tromsø, Norway; Department of Epidemiology, Faculty of Medicine, Prince of Songkla University, Hat Yai, Songkhla 90110, Thailand; Department of Community Medicine and Global Health, Institute of Health and Society, University of Oslo, Post Box 1130 Blindern, 0318 Oslo, Norway

**Keywords:** domestic violence, ever-married women, female genital mutilation/cutting, Health and Demographic Survey, Somalia and Somaliland, wife-beating justification

## Abstract

**Background:**

In Somalia, despite its prohibition, female circumcision persists alongside significant intimate partner violence. This study examines the prevalence of wife-beating justification among Somali women and its link to the perception that female genital mutilation/cutting (FGM/C) is a religious obligation.

**Methods:**

We studied 7726 married Somali women 15–49 y of age from the 2020 Somali Health and Demographic Survey. Using χ^2^ tests and logistic regression, we examined wife-beating justification by covariates and its connection to the perception that FGM/C is a religious obligation.

**Results:**

The prevalence of women justifying wife-beating for any of six reasons was 56.5% (95% confidence interval [CI] 55.3 to 57.6). A higher prevalence of wife-beating justification was found among women 35–49 y of age (59.9% [95% CI 57.8 to 61.9]), without education (57.7% [95% CI 56.5 to 59.0]), rural residents (57.8% [95% CI 56.3 to 59.2]), with lower socio-economic status (60.4% [95% CI 58.7 to 62.1]) and married before age 18 y (58.4% [95% CI 56.7 to 60.1]). Adjusted for covariates, logistic regression analyses indicated a significant association between wife-beating justification and the belief that FGM/C is mandated by religion (adjusted odds ratio 1.40 [95% CI 1.17 to 1.68], p<0.001).

**Conclusions:**

Wife-beating justification is alarmingly common among Somali women and significantly associated with the belief that FGM/C is mandated by religion. Further research is necessary to investigate the drivers behind the acceptance of domestic violence, its impact on women's mental health and well-being and its association with FGM/C acceptance.

## Introduction

Even though the World Health Organization (WHO) considers violence against women a major public health issue and a violation of women's human rights,^1^ in all societies, to a greater or lesser extent, women and girls are subjected to various forms of violence.^2^ Violence against women may result in sexually transmitted infections, unwanted/unplanned pregnancies, abortion,^3^ high-risk fertility behaviors,^4^ traumatic injuries and death.^5^ Much of the violence against women is perpetrated by husbands or intimate partners. The World's Women 2020 report indicates that one in three women, regardless of age, education or income, has experienced physical and/or sexual violence perpetrated by current or former intimate partners during the 12 months before the survey, with 14% reporting physical violence and 6% reporting sexual violence.^6^ Wife-beating is one of the predominant forms of intimate partner violence (IPV) and refers to psychological, sexual and/or physical abuse inflicted by a woman's current or former husband or intimate partner.^7,8^ Although women experience wife-beating in all societies, wife-beating experience varies by continent and country. Sub-Saharan Africa (SSA) has the highest prevalence of IPV against women, including wife-beating, with an overall prevalence of 36% (south 30%, east 39%, west 42% and central 65%).^9^

Despite the extent of violence that women are subjected to, women accept violence perpetrated by a husband against his wife,^10^ i.e. wife-beating. Women who have experienced wife-beating were more likely to justify a husband beating his wife.^11^ This highlights the relationship between women's attitudes about wife-beating and their vulnerability to IPV, suggesting that permissive attitudes may lead to an increased risk of IPV. Wife-beating acceptance among women is more common in Africa and South Asia than in Central and Eastern Europe and Latin America and the Caribbean.^12^ However, the acceptance of physical violence among women by their partners declined by approximately 75% from 2012 to 2019 in countries with available data.^6^

In Somalia, violence against girls and women is widespread and often underreported,^13^ and the ongoing armed conflicts and natural disasters, including droughts and floods, in many parts of the country has created an environment conducive to violence against girls and women.^14^ In 2018, government agencies reported 218 cases of violence against women and girls in Somaliland, 312 cases in Puntland and 400 cases in south-central Somalia.^15^ Somaliland is relatively stable compared with Somalia. Even though the scarcity of data has been an obstacle to fully understanding the occurrence, disparities and impacts of violence against women in Somali regions, international organizations have shown that violence against girls and women is increasing, particularly sexual violence and IPV.^13^

In Somalia, no law punishes perpetrators of domestic violence, and legal protection for survivors is insufficient.^16,17^ In Somaliland, survivors and their advocates rely on prohibitions contained in the 1962 Somali Penal Code.^18^ In addition, according to Somalia's Provisional 2012 Constitution, circumcision of girls is prohibited, but there exists no law that criminalizes female genital mutilation/cutting (FGM/C) or punishes it.^19^ Almost all Somali women undergo FGM/C; 99.2% of women 15–49 y of age (98% in Somaliland) have undergone a form of FGM/C, which is the world's highest prevalence,^20^ and some women believe FGM/C is mandated by religion.^21^ FGM/C is widely practiced in Africa and many countries in the Middle East^22^ and the WHO defines FGM/C as including ‘all procedures that involve the partial or total removal of external genitalia or other injury to the female genital organs (such as stitching of the labia majora or pricking of the clitoris) for non-medical reasons’.^21^

Attitudes and beliefs of both victims and perpetrators toward violence have been considered important for understanding factors that drive and perpetuate domestic violence.^23,24^ In the Somali context, no studies have addressed women's attitudes toward domestic violence and the factors that influence their attitudes toward wife-beating acceptance. Both cultural norms and religious beliefs shape attitudes about FGM/C and IPV in Somalia and Somaliland. While Islam does not explicitly endorse FGM/C, some practitioners cite religious beliefs and cultural norms to perpetuate the practice.^25^ Both FGM/C and IPV are rooted in social and cultural norms, but the relationship between FGM/C and IPV can be theoretically understood through social learning theory.^26^ Learning theory offers a framework to comprehend the acquisition, reinforcement and perpetuation of behaviours associated with IPV and FGM/C within social and cultural contexts. By recognizing the role of the learning process, we can understand how individuals learn behaviours and attitudes related to both FGM/C and IPV within their social and cultural context. For instance, children in households where FGM/C is practiced and/or IPV is normalized might consider these behaviours acceptable, increasing the likelihood that they perpetuate them in their future households and relationships. Research shows that women who have undergone FGM/C are at higher risk of experiencing IPV,^27^ but little is known about the relationship between women's attitudes toward wife-beating and their attitudes toward FGM/C. Hence, understanding the link between women's acceptance of wife-beating and their vulnerability to IPV and cultural practices like FGM/C is crucial for promoting gender equality and preventing violence against women globally. Both practices reflect controlling of women's bodies and perpetuate gender inequality. The present study among 15- to 49-year-old ever-married Somali women aims to estimate the prevalence of Somali women's attitudes towards wife-beating justification; estimate the prevalence of wife-beating acceptance by selected sociodemographic factors, exposure to media and use of the internet; and investigate the association between wife-beating acceptance and the belief that FGM/C is mandated by religion.

## Methods

### Data source and population

The study used data from the 2020 Somali Health and Demographic Survey (SHDS).^28^ The 2020 SHDS was the first of its kind conducted in Somalia and was carried out from 2018 to 2019. Its primary purpose was to provide information on the health and demographic characteristics of the Somali population to direct national development policies and projects.^20^ We accessed data through the website of the Somali National Bureau of Statistics.

The 2020 SHDS was a representative probability household sample that was designed to collect data on housing characteristics, family planning, child health, nutrition, fertility, gender-based violence, female circumcision, chronic disease and knowledge, beliefs and attitudes towards human immunodeficiency virus/acquired immunodeficiency syndrome.^20^ A three-stage stratified cluster sampling design was used with probability proportional to size for the primary and secondary sampling units and systematic sampling of households at the final stage. The data were collected in face-to-face interviews and an ever-married woman's questionnaire was administered to women ages 15–49 y. The total number of households selected for the sample in the SHDS 2020 was 15 870 and 16 715 women were interviewed. The household response rate was 99% (households interviewed divided by households occupied), while the response rate of eligible ever-married women (ages 15–49 y) was 92% (ever-married women interviewed divided by eligible ever-married women). The present study included 7726 ever-married women with complete data on the outcome variable (2948 from Somaliland and 4778 from Somalia).

### Measurements

#### Dependent variable

The dependent variable is whether women justified or refused wife-beating by a husband or intimate partner. The 2020 SHDS used a six-item wife-beating scale. Ever-married women were asked their opinion on whether a husband is justified in hitting or beating his wife for six reasons, with response categories yes, no and don't know: If she wastes resources? If she refuses to have sex with him? If she argues with him? If she neglects household duties? If she neglects the children? If she goes out without telling him?

If a woman accepted wife-beating for at least one of the six reasons mentioned above, she was considered to have a tolerant attitude toward wife-beating. A binary variable was formed (refused wife-beating for all six reasons=0, accepted wife-beating for at least one reason=1). This final dichotomous variable was used as a dependent variable in the statistical analysis.

#### Exposure variable

The exposure variable in the present study is the belief that FGM/C is mandated by religion, which was measured by the question, ‘Do you believe that female circumcision is required by your religion?’ Response categories were yes and no.

#### Sociodemographic variables

The ever-married women were grouped into three age groups: 15–24, 25–34 and 35–49 y. Education had four levels (no education, primary, secondary and higher), but we operationalized education into two groups (no education, primary or higher) as each of the latter categories had small frequencies. The socio-economic status of women's households was measured in wealth quintiles (lowest, middle and highest) based on information on household dwelling, characteristics and access to a variety of consumer goods and services, and is in line with expenditure and income measures (for a detailed discussion, see the Guide to DHS statistics).^29^ Also, the study included work status (whether the respondent is currently working or not) and type of residence (rural, urban). In the 2020 SHDS, the 18 administrative regions of Somalia before the civil war in 1988 were used as a stratification variable alongside the type of residence. For ease of comparison, the region variable was recoded with the two main categories (1=Somaliland, 2=Somalia). Somaliland is currently an independent but not internationally recognized state that includes 5 administrative regions of the former 18 regions of Somalia. The second category (Somalia) includes the other 13 administrative regions. Finally, we included age at first marriage, operationalized into ages (<18=0, ≥18=1).

Television viewing was measured by the question, ‘Do you watch television at least once a week, less than once a week or not at all?’ Radio listening was measured by the question, ‘Do you listen to the radio at least once a week, less than once a week or not at all?’ Lifetime use of the internet was measured by the question, ‘Have you ever used the internet?’ The response categories were yes and no.

### Statistical methods

**Figure 1.**
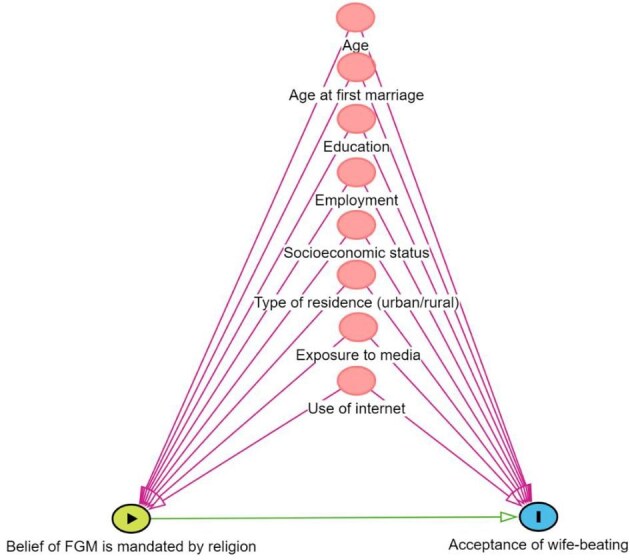
Directed acyclic graph^29^ for the association between the belief that FGM/C is religiously mandated and acceptance of wife-beating. The variables marked in red represent the identified confounders that are adjusted for in the logistic regression analyses.

The directed acyclic graph^30^ presented in Figure 1 summarizes the hypothesized association between wife-beating acceptance and the belief that FGM/C is mandated by religion We identified the following confounders, which will be adjusted for in regression analyses: sociodemographic variables (age, age at first marriage, education, employment, socio-economic status, type of residence and region) and exposure to media and use of the internet. Please note that the proposed association is not a causal or temporal relationship, as the study is based on cross-sectional data.

Inconsistencies and outliers were checked in the data, and to account for the complex sampling design used in the SHDS 2020, the data were weighted before any analysis was carried out. The reliability of the wife-beating scale was checked and the internal consistency of the six-item wife-beating scale used in the SHDS 2020 had a high internal consistency (Cronbach's α=0.94). Descriptive statistics, such as percentages with 95% confidence intervals (CIs), were used to present the demographic background of the respondents and the prevalence of wife-beating acceptance by demographic variables. Bivariate analysis was conducted using the χ^2^ test. To examine the association between the exposure variable (wife-beating acceptance) and the outcome (the belief that FGM/C is mandated by religion), multivariable logistic regression analysis was performed through a complex samples analysis window in SPSS version 26 (IBM, Armonk, NY, USA) to account for the complex sample design. In both bivariate and multivariable analysis, listwise deletion was used, where cases with missing values in at least one of the specified variables were excluded from the analysis. For the complex samples logistic regression model, explanatory variables with a p-value >0.20 were dropped from the final analysis. Additionally, multicollinearity was tested among explanatory variables and the association between categorical variables was assessed using the φ coefficient and Cramer's V. The highest correlation was observed between education and use of the internet (φ=0.432, p<0.001), thus multicollinearity was not considered problematic.^30^ We used SPSS version 26 (IBM) for data management and statistical analysis and p-values <0.05 were considered statistically significant.

## Results

### Background of the respondents

Most participants were 25–34 y of age (42.2%), resided in rural areas (63.3%), had no formal education (83.4%) and were unemployed (92.1%) (Table 1).

**Table 1.** Demographic characteristics of the study population, women in Somalia and Somaliland ages 15–49 y (N=7726)

Characteristics	n	%
Age (years): mean 29.6 (standard deviation 7.7)		
Age groups		
15–24	2156	27.6
25–34	3259	42.2
35–49	2311	30.2
Age at marriage (years)		
<18	3878	53.6
≥18	3353	46.4
Education		
No education	6408	83.4
Primary or higher	1318	16.6
Employment status		
Yes	584	7.9
No	7142	92.1
Socio-economic status		
Lowest	3297	43.7
Middle	2962	38.0
Highest	1467	18.3
Location of residence		
Rural	4312	63.3
Urban	3414	36.7
Region		
Somaliland	2948	40.0
Somalia	4778	60.0

The percentages consider the weighting variable.

### Prevalence of wife-beating acceptance

Table 2 shows the prevalence of women who justified wife-beating under the six provided reasons, with 56.5% endorsing it for at least one reason. The most commonly accepted reasons for wife-beating included neglecting children (41.4%), neglecting household duties (41.3%) and refusing sex with the husband (41.6%). Overall, permissive attitudes were higher among women from Somaliland and those from rural areas.

**Table 2.** Prevalence of reasons for wife-beating justification by region and residence of women ages 15–49 years (N=7726)

	Overall prevalence		Somaliland		Somalia		Urban		Rural	
A husband is justified in beating his wife if she	%	95% CI	%	95% CI	%	95% CI	%	95% CI	%	95% CI
Goes out without telling husband	37.9	36.9 to 39.1	42.8	41.0 to 44.6	34.7	33.3 to 36.1	35.7	33.9 to 37.5	39.2	37.8 to 40.6
Neglects the children	41.4	40.3 to 42.6	47.0	45.2 to 48.8	37.7	36.3 to 39.2	39.8	37.9 to 41.7	42.4	41.0 to 43.8
Neglects the household duties	41.3	40.2 to 42.4	46.4	44.6 to 48.2	37.9	36.4 to 39.3	36.8	35.0 to 38.7	43.9	42.4 to 45.3
Argues with him	40.6	39.5 to 41.7	47.6	45.8 to 49.4	36.0	34.6 to 37.4	37.8	36.0 to 39.7	42.2	40.8 to 43.7
Wastes resources	40.8	39.7 to 42.0	48.1	46.3 to 49.9	36.0	34.6 to 37.4	38.5	36.6 to 40.3	42.2	40.8 to 43.6
Refuses sex with him	41.6	40.4 to 42.7	48.7	46.9 to 50.6	36.8	35.3 to 38.2	38.6	36.8 to 40.5	43.2	41.8 to 44.7
Justified wife-beating at least for one reason	56.5	55.3 to 57.6	59.8	58.0 to 61.6	54.2	52.7 to 55.7	54.3	52.4 to 56.1	57.8	56.3 to 59.2

Percentages consider the weighting variable.

### Prevalence of wife-beating acceptance by sociodemographic variables, exposure to media and use of the internet

The prevalence of wife-beating acceptance was higher among women in the older age groups (35–49 y; 60% [95% CI 57.8 to 61.9]) (Table 3). Regarding other variables, the prevalence of wife-beating acceptance was greater among women with no education, with lower socio-economic status and in the Somaliland region. However, wife-beating acceptance had no significant association with age at first marriage, the woman's employment status, type of residence (rural/urban) and exposure to media (TV viewing, radio listening) (p>0.05).

**Table 3.** Prevalence of wife-beating justification and χ^2^ tests of independence by the sociodemographic factors exposure to media and use of the internet (N=7726)

	Women who justified wife-beating for at least one reason				
Characteristics	n	%	95% CI		p-Value
Age group (years)					
15–24	1218	54.9	52.7	57.1	0.026
25–34	1820	55.0	53.0	56.8	
35–49	1348	59.9	57.8	61.9	
Age at first marriage (years)					NS
<18	2272	58.4	56.7	60.1	
≥18	2104	54.9	53.3	56.9	
Education					
No education	3490	57.7	56.5	59.0	0.001
Primary or higher	602	50.1	47.3	52.9	
Employment status					
Yes	352	57.7	53.6	61.7	NS
No	4044	56.4	55.2	57.5	
Socio-economic status					
Lowest	1916	60.4	58.7	62.1	0.002
Middle	1506	54.8	52.8	56.6	
Highest	669	50.7	47.9	53.3	
Location of residence					
Rural	2540	57.8	56.3	59.2	NS
Urban	1846	54.3	52.4	56.1	
Region					
Somaliland	1732	59.8	58.0	61.6	0.020
Somalia	2359	54.2	52.7	55.7	
Frequency of TV viewing					
≤once a week	445	55.0	51.6	58.4	NS
Not at all	3647	56.7	55.5	57.9	
Frequency of radio listening					
≤once a week	423	59.2	55.6	62.8	NS
Not at all	3669	56.2	55.0	57.4	
Ever used the internet					
Yes	313	43.4	39.7	46.9	0.000
No	3778	57.9	56.7	59.1	

Calculations are based on the complex sample design and sampling weights of the 2020 SHDS.

NS: not significant.

### Wife-beating acceptance and belief that FGM/C is mandated by religion

The multiple logistic regression results in Table 4 show a significant association between the belief that FGM/C is mandated by religion and wife-beating acceptance for at least one reason (adjusted odds ratio 1.40 [95% CI 1.17 to 1.68], p<0.001).

**Table 4.** Association of the belief that FGM/C is religiously mandated and accepting wife-beating for at least one reason in women from Somalia and Somaliland ages 15–49 y (N= 6662)

Explanatory variables	OR (95% CI)	p-Values	Adjusted OR (95% CI)	p-Value
FGM/C is required by religion				
Yes	1.47 (1.31 to 1.64)	0.000	1.40 (1.17 to 1.68)	0.000
No (ref)	1		1	

Odds ratios (ORs) for belief in FGM/C is mandated by religion were adjusted for age, education, use of the internet, socio-economic status, location of residence and region in the final logistic regression model leaving out factors with a p-value >0.20.

## Discussion

The overall prevalence of wife-beating acceptance for at least one of the provided six reasons among Somali ever-married women was 57%, while there were minor differences in the prevalence of each of the six reasons for acceptance of wife-beating at 40–42%.

The prevalence of wife-beating acceptance was higher in some sociodemographic subgroups: older age, no formal education, low socio-economic status, living in Somaliland as compared with south-central Somalia and among those who never use the internet (p<0.05). There was a significant association between the belief that FGM/C is religiously mandated and acceptance of wife-beating for at least one reason (p<0.001).

The percentage of Somali ever-married women who justified wife-beating for the given reasons was higher than reported in a recent study, which included DHS data collected from 2008 through 2020 in 30 SSA countries.^32^ In another older DHS study with data collected between 2003 and 2007 that included 17 SSA countries, women from Mali, Democratic Republic of Congo, Chad and Guinea had the highest prevalence of wife-beating justification, all >60%. Like Somalia, these countries are characterized by political instability and have a history of armed conflicts.^33^ Also, the acceptance of violence against women is deeply rooted in cultural norms, gender roles, religious beliefs and socio-economic conditions. Countries with a high prevalence of wife-beating acceptance often exhibit similar patterns in these factors.

Our finding of a higher prevalence of wife-beating acceptance among women who never use the internet is in agreement with other studies.^34,35^ Likewise, we confirmed findings from previous studies that the prevalence of wife-beating acceptance is higher among women with no education, living in rural areas and with low socio-economic status.^36–38^ Although Somaliland is relatively more stable and has lower rates of IPV,^17^ wife-beating acceptance was higher among women from Somaliland than those from Somalia. In the present study, we report that women who believe that FGM/C is mandated by religion have increased odds of accepting wife-beating. As far as the authors are aware, this is the first study examining the association between the belief that FGM/C is mandated by religion and wife-beating acceptance in the Somali context.

In light of the cultural context of Somalia, the significant association we found between attitudes about FGM/C and attitudes about wife-beating reflect deeply interconnected cultural factors. For instance, the patriarchal nature of Somali society and gender roles influences women's lives^39^ and may influence women's attitudes toward both FGM/C and wife-beating. Women's behaviour and expectations are greatly influenced by their role in Somali society, where married women are expected to be under the authority of their husbands. Similarly, religious teachings and perceived religious mandates influence women's attitudes, as women who believed FGM/C is mandated by religion had permissive attitudes toward wife-beating. Therefore, understanding Somali women's attitudes about both FGM/C and wife-beating and their relationship requires an approach that includes all these cultural factors. Also, government, civil society and international intervention programs that aim to fight against FGM/C and IPV must consider these cultural factors in their program designs.

Illiteracy among women is very high, particularly women from rural areas, thus limited access to education may significantly affect their opportunities, perspectives and attitudes. Women with no education are more likely to accept wife-beating. The strengths of the current study include the use of a representative probability sample and the standardized questionnaires of the Demographic and Health Surveys Program, which makes results comparable across countries.

However, the study has limitations. First, the results are based on cross-sectional data, which cannot establish a temporal relationship between exposure (FGM/C mandated by religion) and outcome (acceptance of wife-beating). Second, given the sensitivity of the topic within the Somali cultural context, women's responses may be influenced by cultural bias. Somali men and women often do not speak out against husbands’ rights to use violence and protect family honor.^40^ Furthermore, in Somali culture, marriage is not only between individuals, but also between families.^41^ Therefore, women's attitudes toward wife-beating might be heavily influenced by cultural norms.

## Conclusions and implications

The prevalence of wife-beating acceptance was high, and was greater among uneducated women, those who never used the internet, women from poor households, those from rural areas and those living in the Somaliland region as compared with Somalia. There is a significant association between the belief that FGM/C is mandated by religion and wife-beating acceptance. While attitudes and behavioural shifts often require considerable time, the outcomes of this study underscore the importance of integrating formal and informal educational initiatives within interventions or rights awareness programs conducted by authorities or non-governmental organizations. Our findings call for the need for multiple strategies to reduce FGM/C and IPV, such as community education and awareness, empowerment programs, legislative measures and research and data collection. Through the utilization of a mix of these approaches, communities and governments can strive to decrease the occurrence of FGM/C and IPV, advance gender equality and foster safer and healthier environments for women and girls. It is essential to track changes in attitudes toward violence against women over time and evaluate the lasting impact of economic and educational empowerment efforts in combating IPV and FGM/C. Thus future studies could examine the effectiveness of various economic and educational programs, formal and informal alike, in reducing acceptance of domestic violence and promoting gender equality. Additionally, gaining a deeper understanding of how attitudes shift can aid in designing more effective interventions. Exploring the role of religious institutions and influential individuals in shaping perceptions of violence against women also holds promise for further investigation.

## Data Availability

The data used for this article is publicly accessible from the Somali Bureau of Statistics website (https://microdata.nbs.gov.so/index.php/catalog/50).
